# Evaluación de 18 indicadores de calidad del Programa de Garantía Externa de la Calidad de Preanalítica de la Sociedad Española de Medicina de Laboratorio (SEQC^ML^)

**DOI:** 10.1515/almed-2022-0036

**Published:** 2022-06-08

**Authors:** Andrea Caballero, Rubén Gómez-Rioja, Montserrat Ventura, Ma Antonia Llopis, Josep Miquel Bauça, Carolina Gómez-Gómez, Itziar Marzana, Mercedes Ibarz

**Affiliations:** Comisión de Calidad Extra-analítica de la Sociedad Española de Medicina de Laboratorio (SEQCML), Departamento de Bioquímica Clínica, Laboratorio Echevarne, Sant Cugat del Vallés, España; Comisión de Calidad Extra-analítica de la Sociedad Española de Medicina de Laboratorio (SEQC^ML^), Servicio de Análisis Clínicos, Hospital La Paz-Cantoblanco-Carlos III, Madrid, España; Comisión de Calidad Extra-analítica de la Sociedad Española de Medicina de Laboratorio (SEQC^ML^), Responsable Laboratorios Clínicos, Instituto Catalán de la Salud (ICS), Badalona, España; Comisión de Calidad Extra-analítica de la Sociedad Española de Medicina de Laboratorio (SEQC^ML^), Departamento de Laboratorio Clínico, Hospital Universitario Arnau de Vilanova, IRBLleida, Lleida, España; Comisión de Calidad Extra-analítica de la Sociedad Española de Medicina de Laboratorio (SEQC^ML^), Servicio de análisis clínicos, Hospital Universitario Son Espases, Palma, España; Comisión de Calidad Extra-analítica de la Sociedad Española de Medicina de Laboratorio (SEQC^ML^), Departamento de Laboratorio Clínico, Universidad Hospital Germans Trias I Pujol, Badalona, Barcelona, España; Comisión de Calidad Extra-analítica de la Sociedad Española de Medicina de Laboratorio (SEQC^ML^), Programas de Garantía Externa de la Calidad, Sociedad Española de Medicina de Laboratorio, Barcelona, España; Comisión de Calidad Extra-analítica de la Sociedad Española de Medicina de Laboratorio (SEQC^ML^), Unidad extraanalítica, Laboratorios Hospital Universitario Cruces, Baracaldo, Vizcaya, España

**Keywords:** fase preanalítica, especificaciones de calidad, indicadores de calidad, programas de garantía externa de la calidad

## Abstract

**Objetivos:**

la mayor parte de los errores en medicina del laboratorio se producen durante la fase preanalítica y postanalítica del proceso analítico total (PAT). En 2014, la Sociedad Española de Medicina de Laboratorio (SEQC^ML^) lanzó el Programa de Garantía Externa de la Calidad (EQA) de Preanalítica, con el propósito de ofrecer una herramienta para mejorar la calidad de la fase preanalítica. El objeto del presente estudio es evaluar la evolución de los indicadores de calidad (IC) y la comparabilidad de las especificaciones de calidad de la fase preanalitica (EC) con otros programas EQA.

**Métodos:**

en el programa de la SEQC^ML^, los participantes registraron el número de rechazos por cada tipo de muestra y por causa de rechazo. El cálculo de los percentiles se realizó a partir de los datos obtenidos en el periodo 2014–2017. Posteriormente, se revisaron dichos percentiles con los datos obtenidos en los años 2018 y 2019. Se calcularon los percentiles 25, 50, 75 y 90 de cada serie, estableciendo las medias como especificaciones. Estas especificaciones se compararon con los resultados de otros programas.

**Resultados:**

en general las especificaciones con respecto a los IC se mantuvieron estables o mejoraron en el periodo 2018–2019, por ejemplo, se produjo un descenso significativo en el número de muestras de suero con un índice hemolítico ≥0,5 g/L. Sin embargo, este descenso no se observó en el manejo de muestras de EDTA y citrato, posiblemente debido a una mejor capacidad de detección de la hemólisis. Las especificaciones para los IC del grupo de trabajo *Laboratory Errors and Patient Safety* de la IFCC y del programa *Key Incident Management and Monitoring System* (KIMMS) de la RCPA mostraron resultados comparables, lo que confirma la validez de las especificaciones establecidas.

**Conclusiones:**

las especificaciones obtenidas son una herramienta útil para la intercomparación, así como para identificar aquellos procesos de la fase preanalítica que son prioritarios mejorar.

## Introducción

Se ha demostrado que la mayoría de los errores en la medicina de laboratorio se producen en las fases preanalítica y postanalítica del proceso analítico total (PAT), lo que a su vez, se traduce en un mayor riesgo para el paciente [1]. Aunque el concepto del ciclo “cerebro a cerebro” en la medicina de laboratorio se describió hace más de 45 años [2], conseguir concienciar y alcanzar un consenso sobre el importante papel que juegan los elementos extra-analíticos en la calidad analítica es un logro mucho más reciente [3, 4]. Todos los pasos del PAT se deben controlar y evaluar de forma continua, con el fin de garantizar que se proporcionan resultados analíticos de la máxima calidad [5].

La incorporación de sistemas internos de control de calidad [6] y la participación en programas de garantía externa de la calidad (EQA, por sus siglas en inglés) son herramientas esenciales a la hora de lograr mejorar dichos procesos de manera continua [7, 8]. Los indicadores de calidad (IC), considerados una parte de la estrategia de mejora de la calidad analítica, han demostrado ser una herramienta adecuada a la hora de controlar y mejorar la calidad de la fase extra-analítica [9, 10]. De hecho, según la ISO 15189:2012, la identificación y empleo de IC efectivos en todas las fases del PAT es un requisito imprescindible para que un laboratorio obtenga la acreditación [8], debiendo comprobarse la totalidad del proceso de examen, incluidos los procedimientos previos y posteriores del mismo [11].

En las últimas décadas, se han desarrollado en distintos países tres tipos de estrategias de programas EQA de la fase preanalítica (Tabla 1) [12]: tipo I: registro de procedimientos; tipo II: distribución de muestras que simulan errores y tipo III: registro de errores/eventos adversos. En el tipo III, el organizador del programa EQA debe también armonizar los IC para poder realizar una comparación válida de los índices de error y proporcionar especificaciones de la calidad (EC). En tres de los programas que se corresponden con este tipo de estrategia se han establecido especificaciones derivadas del estado del arte, son los programas de la *International Federation of Clinical Chemistry and Laboratory Medicine* (IFCC) [13], la *Royal College of Pathologists of Australasia* (RCPA) [14] y la Sociedad Española de Medicina de Laboratorio (SEQC^ML^).

**Tabla 1:** Programas de garantía externa de la calidad (EQA) de la fase preanalítica (modificado por Kristensen y col. 2014 [12]).

Tipo	Programa	Organización	País	Año de Inicio	Web
Tipo I	Programa de garantía externa de la calidad de la fase preanalítica	SEQC^ML^	España	2001^a^	
	*NOKLUS preanalytical EQAs*	NOKLUS^b^	Noruega	2008	http://www,noklus,no
	*Four Preanalytical EQAs Programmes* (formación en línea)	INSTAND e,V	Alemania	2011	https://www,instand-ev,de/en
	Preanalytics programme in clinical chemistry, anatomic pathology, microbiology, urine, and blood sample collection and POCT^c^	Labquality	Finlandia	2014	https://www,labquality,fi/en/eqas/?lang=en
Tipo II	Preanalytical serum indices scheme	WEQAS^d^	Reino Unido	2010	http://www,weqas,com
	Programa de garantía externa de la calidad para índices séricos	SEQC^ML^	España	2018	https://www,seqc,es/es/programas-garantia-calidad
	*EQA Programe addressing the important preanalytical workflows applied to personalized medicine*	SPIDIA^e^ y SPIDIA4P	Asociados de Dinamarca, Reino Unido, Suiza, Suecia, Italia, Austria, Luxemburgo, Francia, Países Bajos y España	2011	https://www,spidia,eu
Tipo III	*The Q-Track Programmes*	CAP^f^	EE,UU,	1998	https://www,cap,org/laboratory-improvement/quality-management-Programas
	*Key Incident Monitoring and Management Systems Quality Assurance (KIMMS)*	RCPA	Australia	2009	https://rcpaqap,com,au
	Programa de garantía externa de la calidad de la fase preanalítica	SEQC^ML^	España	2014	https://www,seqc,es/es/programas-garantia-calidad
	*NOKLUS preanalytical EQAs*	NOKLUS	Noruega	2017	http://www,noklus,no
	*UK NEQAS Pre- and Post-Analytical Quality Monitoring Service*	UK NEQAS^g^	Reino unido	2019	https://birminghamquality,org,uk/prepq/
	*Model of Quality Indicators of WG-LEPS*	IFCC	A nivel mundial	2017	https://www,ifcc,org/ifcc-education-division/working-groups-special-projects/laboratory-errors-and-patient-safety-wg-leps/quality-indicators-project/

^a^Finalizado en 2013, ^b^Norwegian Organization for Quality Improvement of Laboratory Examinations, ^c^point-of-care testing, ^d^Wales External Quality Assurance Scheme, ^e^Standardization and Improvement of Generic Pre-analytical Tools and Procedures for *In Vitro* Diagnostics, ^f^College of American Pathologists, ^g^United Kingdom National External Quality Assessment Service.

Una EC es un valor cuantitativo que, cuando se excede, requiere la adopción de medidas correctivas [15]. De acuerdo con la I Conferencia Estratégica de la *European Federation of Clinical Chemistry and Laboratory Medicine* (EFLM) celebrada en Milán en 2015, las definiciones de EC para la fase extra-analítica deberán seguir los mismos modelos jerárquicos que los de las EC analíticas, esto es, resultados clínicos, variabilidad biológica y estado del arte [9, 15–18]. No obstante, en esta fase del PAT, el estado del arte es el criterio más fácil de aplicar [1, 10]. También se propusieron tres niveles de EC, siguiendo el modelo desarrollado por *Fraser y col*. para las EC analíticas [19]: primer nivel: resultados individuales por debajo del percentil 25 (p25) de la distribución de valores, lo que representa la mejor prestación. Segundo nivel: valor en el percentil 50 (p50), que representa la prestación más común y frecuente y el tercer nivel: el percentil 75 (p75), que representa el peor desempeño [17]. Así mismo, se pueden emplear otros niveles, como el percentil 10 (p10), para designar a los “laboratorios excelentes”, estando la mayoría de los laboratorios entre los percentiles 10 y 90 (p90) y considerando los laboratorios con valores de EC fuera del p90 como los de “peor desempeño”. Por otro lado, se puede emplear un modelo de un solo límite, establecido en el p25, para determinar si la prestación es aceptable o inaceptable [20].

En 1998, la Comisión de Calidad Extra-analítica de la SEQC^ML^ lanzó un programa piloto de garantía externa de la calidad de la fase preanalítica de tipo I, que se consolidó en 2001. Hasta 2013, el programa se centró en analizar las causas de los rechazos de muestras de sangre y orina. Los resultados obtenidos durante los primeros cinco años del programa (2001–2005) se publicaron en 2006 para las muestras de orina [21] y en 2008 para las muestras de sangre [22], y en 2016 se publicó una evaluación del periodo completo de los 13 años [7]. Este primer programa aportó información sobre los principales tipos de errores que se produjeron en el proceso preanalítico en los laboratorios españoles, que fueron la hemólisis, seguido de las muestras de sangre no recibidas o coaguladas y la no recepción en las muestras de orina.

En 2014, el programa se reconvirtió a tipo III para mejorar la accesibilidad de los usuarios, facilitando la recogida y el envío de datos, así como para mejorar la robustez del mismo, incrementando el periodo de tiempo en el que los laboratorios podían registrar los rechazos. Desde entonces, se han procesado y analizado los datos de los laboratorios participantes estableciendo los p25, p50, p75 y p90 como especificaciones. El objetivo principal de este programa es ofrecer una herramienta sencilla para detectar los errores más frecuentes en la fase preanalítica, así como promover la intercomparación de resultados y fomentar la mejora continua [23], cuando los resultados de los IC superan las especificaciones sugeridas, se insta al laboratorio a adoptar medidas correctivas [15].

El objetivo de este estudio es revisar la coherencia y evolución de los IC inicialmente establecidos tras un periodo de cuatro años (2014–2017), y posteriormente revisados bienalmente (2018–2019), y evaluar la comparabilidad de las EC con los programas EQA del grupo de trabajo *Laboratory Errors and Patient Safety* (WG-LEPS) de la IFCC y el programa *Key Incident Management and Monitoring System* (KIMMS) de la RCPA.

## Materiales y métodos

### Diseño del programa

En el programa de Preanalítica de la SEQC^ML^, los participantes deben registrar los rechazos de los principales tipos de muestras, y sus causas, producidos a lo largo de un mes, cuatro veces al año. A continuación, se calculan los IC, que se expresan como porcentaje de rechazos respecto al número total de pruebas más frecuentes para cada tipo de muestra (creatinina para el suero, hemograma para las muestras de sangre total EDTA y tiempo de protrombina para las de plasma citrato). Puntualmente, los IC se refieren al número total de peticiones, si no existe un tipo principal de espécimen o de prueba para dicho espécimen. En la Tabla 2 se muestran las definiciones de los IC, así como el valor medio de los p25, p50, p75 y p90 empleados para calcular las EC.

**Tabla 2:** Lista de indicadores de calidad (IC) del programa de garantía externa de la calidad de la fase preanalitica de la SEQC^ML^ y sus fórmulas de cálculo. Especificaciones de calidad (EC) calculadas a partir de los resultados del programa para el periodo 2014–2017 y 2018–2019, se muestran las medianas de la diferencia entre periodos (negativa cuando se produce una mejora en el percentil p50 y positiva cuando se produce un empeoramiento).

Tipo de IC	IC	Fórmula	EC 2014–2017				EC 2018–2019				Diferencias medianas
			p25	p50	p75	p90	p25	p50	p75	p90	
Rechazos generales	PRE-01	Rechazos totals/Peticiones totales (%)	1,409	2,266	3,272	4,695	1,393	2,182	3,215	4,608	−0,084
	PRE-02	Muestras sin etiqueta/Peticiones totales (%)	0,000	0,009	0,041	0,086	0,000	0,014	0,049	0,102	0,005
	PRE-03	Muestras mal identificadas/Peticiones totales (%)	0,001	0,010	0,030	0,068	0,000	0,009	0,020	0,045	−0,001
Rechazos muestras de suero	PRE-04	Total rechazos de muestras de suero/Pruebas de creatinina (%)	0,452	1,091	2,224	3,534	0,418	0,853	1,737	3,696	**−0,238** ^**a**^
	PRE-05	Muestras de suero no recibidas/Pruebas de creatinina (%)	0,102	0,188	0,328	0,678	0,104	0,210	0,350	0,616	0,022
	PRE-06	Muestras de suero hemolizadas/Pruebas de creatinina (%)	0,222	0,718	1,791	2,876	0,146	0,483	1,397	3,365	**−0,235** ^**a**^
	PRE-07	Muestras de suero insuficiente/Pruebas de creatinina (%)	0,004	0,037	0,107	0,232	0,003	0,035	0,111	0,252	−0,002
Rechazos muestras de sangre total EDTA	PRE-08	Total rechazos de muestras de sangre total EDTA/Hemogramas (%)	0,288	0,481	0,766	1,143	0,317	0,519	0,769	1,066	**0,038** ^**a**^
	PRE-09	Muestras de sangre total EDTA no recibidas/Hemogramas (%)	0,134	0,248	0,414	0,751	0,167	0,266	0,401	0,624	0,018
	PRE-10	Muestras de sangre total EDTA insuficientes (%)/Hemogramas (%)	0,005	0,026	0,068	0,146	0,005	0,022	0,062	0,113	−0,004
	PRE-11	Muestras de sangre total EDTA coagulada/Hemogramas (%)	0,072	0,150	0,252	0,443	0,087	0,162	0,288	0,456	0,012
Rechazos muestras de coagulación plasma citrato	PRE-12	Total rechazos de muestras de coagulación plasma citrato/Pruebas de protrombina (%)	0,811	1,748	2,863	4,734	0,937	1,887	3,298	5,471	0,139
	PRE-13	Muestras de coagulación plasma citrato no recibidas/Pruebas de protrombina (%)	0,371	0,833	1,565	2,273	0,388	0,743	1,468	2,441	−0,090
	PRE-14	Muestras de coagulación plasma citrato insuficientes/Pruebas de protrombina (%)	0,112	0,430	1,028	2,028	0,150	0,465	1,230	2,629	0,035
	PRE-15	Muestras de coagulación plasma citrato coaguladas/Pruebas de protrombina (%)	0,015	0,146	0,381	0,822	0,042	0,204	0,474	0,920	0,058
	PRE-16	Muestras de coagulación plasma citrato hemolizadas/Pruebas de protrombina (%)	0,000	0,000	0,077	0,384	0,000	0,006	0,095	0,557	**0,006** ^**a**^
Rechazos muestras de orina	PRE-17	Muestras de orina no recibidas/Peticiones totales (%)	0,351	0,804	1,359	2,017	0,393	0,877	1,370	1,869	0,073
Calidad de la muestra.	PRE-18	Muestras de suero con índice hemolítico ≥0,5 g/L/Muestras totales de suero con cálculo de índice hemolítico (%)	0,852	1,861	3,399	5,400	0,758	1,503	2,859	4,988	**−0,358** ^**a**^

^a^Significación estadística para el test de la mediana (p<0,05).

### Definición de rechazo

Un “rechazo” se produce cuando no se puede entregar uno o varios de los resultados de una petición debido a errores preanalíticos (cuando el espécimen no cumple los criterios de aceptabilidad del laboratorio, no se realiza o no se proporcionan los resultados de una prueba analítica). El rechazo puede ser global, lo que invalida todas las pruebas solicitadas, o puede afectar únicamente a una prueba en concreto, lo que permite emitir los resultados de otras pruebas.

En los Sistemas Informáticos de Laboratorio (SIL), las pruebas se suelen agrupar en peticiones o solicitudes. En algunos SIL, las peticiones deben ir asociadas a un único tipo de espécimen o muestra, aunque en España es habitual que permitan incluir en una sola petición pruebas analíticas para diferentes tipos de muestras. Por esta razón, en el programa se deben registrar los rechazos por tipo de muestra analizada, esto es, la prueba rechazada se refiere al tipo de espécimen/contenedor en el que se ha realizado. Los cuatro tipos de muestras incluidas en el programa corresponden a los especímenes más comunes en el laboratorio: suero (ya que en los laboratorios españoles es más común emplear suero que plasma en los análisis bioquímicos, y normalmente empleando tubos con gel separador [24]), sangre total EDTA, plasma citrato y orina de micción espontánea. En la Tabla 2 se muestran las fórmulas de cálculo (numerador y denominador) para los diferentes IC.

### Especificaciones de calidad

**Figura 1:**
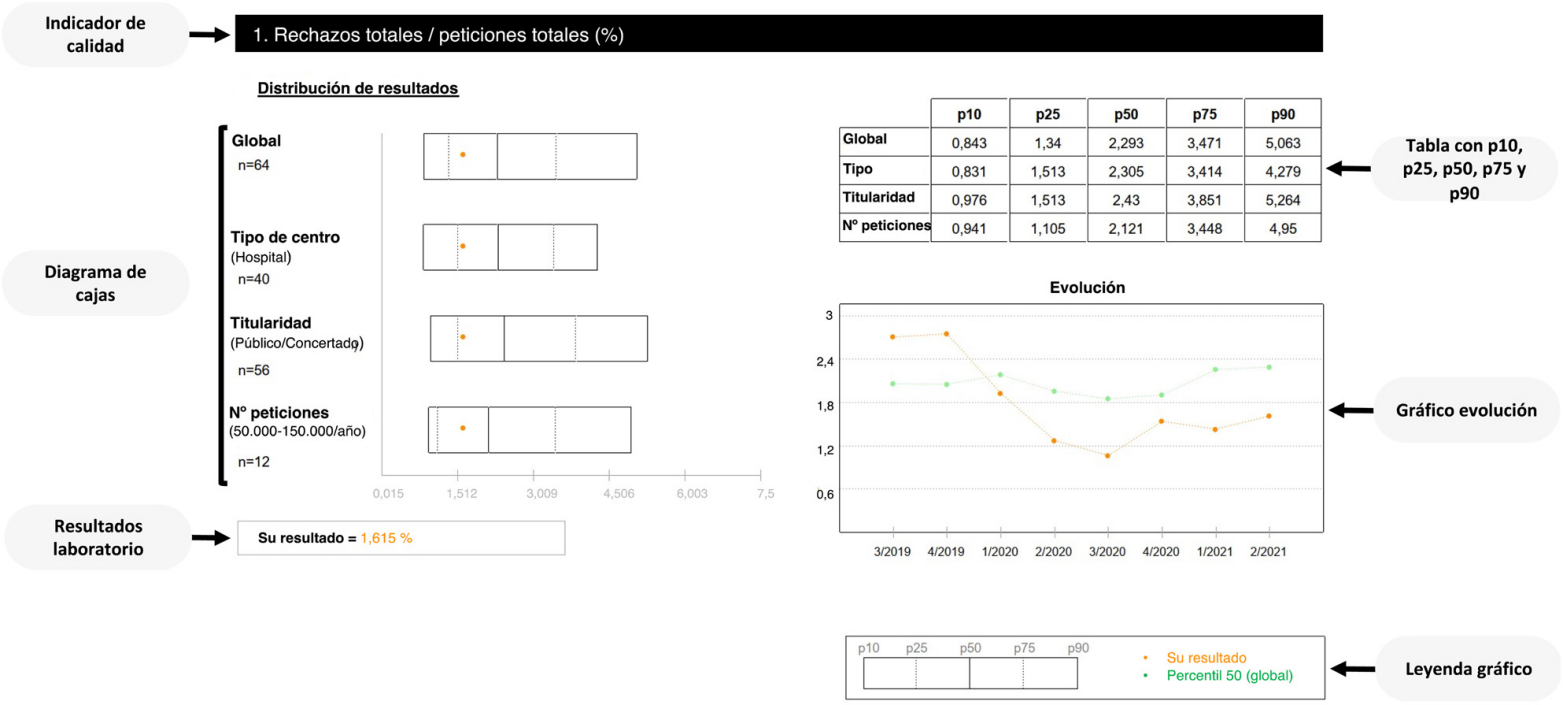
Ejemplo de informe de resultados para el indicador 1 (PRE-01) del programa de garantía externa de la calidad preanalítica de la SEQC^ML^. Se muestran cuatro diagramas de cajas, en los que se compara el resultado del indicador con: (1) Global; percentiles calculados de forma global para todos los laboratorios, (2) Tipo de centro; percentiles calculados para el mismo tipo de centro (Hospital, hospital + atención primaria o laboratorio independiente). (3) Titularidad; percentiles calculados para el mismo tipo de laboratorio (privado o público/concertado). (4) Nº de peticiones; percentiles calculados para los laboratorios con el mismo número de peticiones (25,000–50,000/año, 50,000–150,000/año, 150,000–300,000/año, 300,000–600,000/año o >600,000/año).

Tras procesar los datos extraídos de los informes de cada participante (cuatro veces al año) se compararon los resultados de cada laboratorio con respecto a los p25, p50, p75 y p90 derivados de la distribución de todos los resultados de la serie. La Figura 1 muestra un ejemplo de resultados del programa para un IC.

Las EC del programa, calculadas inicialmente a partir de los datos correspondientes al periodo 2014–2017, se recalcularon con los datos de los años 2018–2019, empleando la media de los percentiles de ocho rondas para establecer las especificaciones de la siguiente manera: p25 como la prestación óptima, p50 como la deseable, p75 como la mínima y también p90, ya que participar voluntariamente en un programa EQA y mantenerse en el 90% central de la distribución de resultados se puede considerar una prestación razonable, de manera análoga a los programas de bioquímica donde no existen especificaciones de nivel superior. Los resultados obtenidos se compararon con los del periodo anterior mediante el test de la mediana. Para los indicadores que se observó una mejoría significativa, se adoptaron las nuevas especificaciones.

### Análisis de datos

Todos los análisis estadísticos se realizaron con el programa IBM SPSS v21 (test de la mediana). El nivel de significación estadística se estableció para un valor p<0,05.

## Resultados

**Figura 2:**
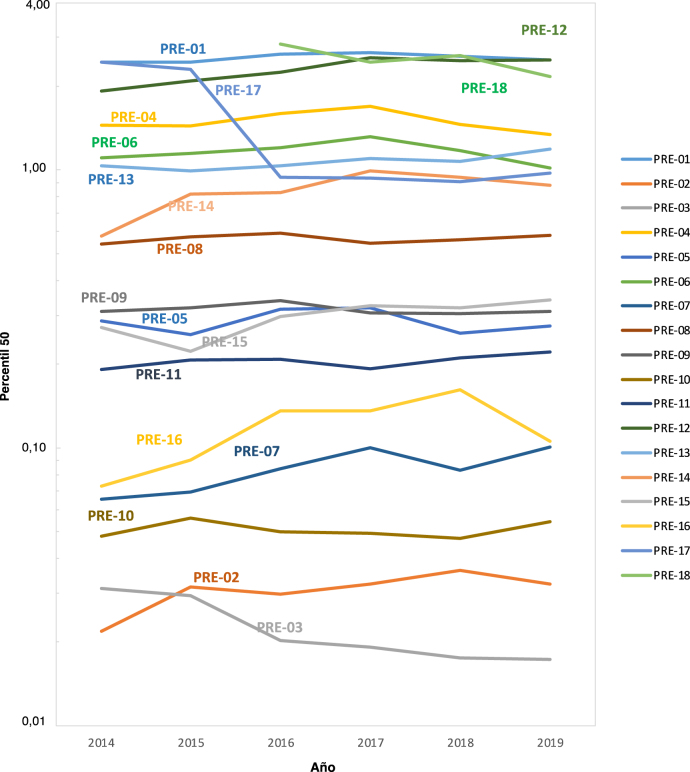
Valores anuales medios de p50 para cada uno de los indicadores de calidad (IC) entre 2014 y 2019.

En el primer periodo (2014–2017) participaron 63 laboratorios, frente a los 72 que participaron en el siguiente periodo de dos años 2018–2019. En la Tabla 2 se muestran las EC para los dos periodos. Se observa un descenso en PRE-04: Nº total de rechazos de muestras de suero, PRE-06: Muestras de suero hemolizadas y PRE-18: Muestras de suero con índice hemolítico ≥0,5 g/L, así como un incremento en PRE-08: Nº total de rechazos de muestras de sangre total EDTA y en PRE-16: Muestras hemolizadas de plasma citrato para pruebas de coagulación (Tabla 2 y Figura 2). El resto de IC se muestran muy estables, con una variabilidad entre periodos inferior al 0,090. La Tabla S1 del Material Suplementario incluye las medias, desviación estándar, coeficientes de variación (CV) y número de envíos de resultados, en cada percentil (p25, p50, p75 y p90).

En la Tabla 3 se incluyen las especificaciones del programa de la SEQC^ML^ comparadas con las establecidas por la IFCC (con 59 laboratorios participantes) y en el programa KIMMS (con 60 laboratorios participantes), así como el CV con respecto al p50 para el periodo completo (2014–2019), siendo este comparable con el CV del programa KIMMS. Las EC para aquellos indicadores con correspondencia entre programas, a pesar de los diferentes denominadores, muestran que el p50 de las muestras mal identificadas es menor en el programa de la SEQC^ML^ que en el de los programas KIMMS y de la IFCC. Cabe señalar que el número de muestras con errores de identificación fue muy bajo en los tres programas (≤0,100). Con respecto al número total de muestras no recibidas, el valor del p50 fue superior en el programa KIMMS que en el de la IFCC, y menor que en el programa de la SEQC^ML^. No obstante, teniendo en cuenta el tipo de espécimen, y según el programa de la SEQC^ML^, las muestras no recibidas más frecuentemente en los laboratorios españoles fueron las muestras de orina (0,877%), seguidas de las muestras de plasma citrato (0,743%), las muestras de sangre total EDTA (0,266%) y las muestras de suero (0,210%). El IC de muestras hemolizadas, la principal causa de rechazo de las muestras de suero, es muy similar a los indicados por la IFCC, siendo inferiores a los de KIMMS. Se observa la misma tendencia con respecto al número de muestras insuficientes o coaguladas. En relación a los CV, en el programa de la SEQC^ML^ se obtuvieron valores superiores a los del programa KIMMS, mostrando mayor variabilidad en los laboratorios que participaron en este programa.

**Tabla 3:** Comparación de las especificaciones de calidad (EC) del programa de la SEQC^ML^ con las de los IC del *Working Group Laboratory Errors and Patient Safety* (WG-LEPS) de la *International Federation of Clinical Chemistry and Laboratory Medicine* (IFCC) [13] y del programa *Key Incident Management and Monitoring System* (KIMMS) de la *Royal College of Pathologists Australasia* (RCPA) [14].

SEQC^ML^ (2018–2019)						IFCC (2108)					KIMMS (2015–2108)		
QI		p25 (CI 95%)	p50 (CI 95%)	p75 (CI 95%)	CV, %^a^	IC		p25 (IC 95%)	p50 (IC 95%)	p75 (IC 95%)	IC	Media	CV, %
PRE-01	Rechazos totales/Peticiones totales, %	1,393	2,182	3,215	8,6		NE				NE		
		(1,279–1,506)	(2,102–2,261)	(3,005–3,426)									
PRE-02	Muestras sin etiqueta/Peticiones totales, %	0,000	0,014	0,049	59,2		NA				Sin etiqueta	0,084	10
		(0,000–0,000)	(0,010–0,018)	(0,043–0,055)									
PRE-03	Muestras mal identificadas/Peticiones totales, %	0,000	0,009	0,020	33,8	Pre-MisR	Porcentaje de: Nº de peticiones mal identificadas/Nº total de peticiones	0,010	0,025	0,070	Discrepancia de ID	0,100	19
		(0,000–0,001)	(0,006–0,011)	(0,018–0,023)				(0,000–0,010)	(0,020–0,030)	(0,054–0,100)			
PRE-04	Nº total de rechazos de muestras de suero/Pruebas de creatinina, %	0,418	0,853	1,737	16,9		NE				NE		
		(0,395–0,441)	(0,785–0,920)	(1,523–1,951)									
PRE-05	Muestras de suero no recibidas/Pruebas de creatinina, %	0,104	0,210	0,350	12,9	Pre-NotRec	Porcentaje de: Nº de muestras no recibidas/Nº total de muestras	0,090	0,190	0,889	Muestras no obtenidas	0,317	6
		(0,094–0,114)	(0,199–0,221)	(0,325–0,375)				(0,060–0,100)	(0,140–0,295)	(0,602–1,470)			
PRE-06	Muestras de suero hemolizadas/Pruebas de creatinina, %	0,146	0,483	1,397	21,2	Pre-HemR	Porcentaje de: Nº de muestras rechazadas por hemólisis/Nº total de muestras en las que se ha medido la presencia hemólisis	0,049	0,435	0,882	Muestra hemolizada	0,770	7
		(0,103–0,189)	(0,029–0,041)	(1,186–1,607)				(0–0,165)	(0,300–0,500)	(0,587–1,410)			
PRE-07	Muestras de suero insuficientes/Pruebas de creatinina, %	0,003	0,035	0,111	26,0	Pre-InsV	Porcentaje de: N^a^ de muestras con volumen insuficiente/Nº total de muestras	0,010	0,030	0,110	Muestra insuficiente	0,165	9
		(0,001–0,005)	(0,029–0,041)	(0,099–0,123)				(0,006–0,011)	(0,022–0,040)	(0,079–0,130)			
PRE-08	Nº total de rechazos de muestras de sangre total EDTA/Hemogramas, %	0,317	0,519	0,769	12,1		NE				NE		
		(0,298–0,336)	(0,488–0,549)	(0,741–0,797)									
PRE-09	Muestras de sangre total EDTA no recibidas/Hemogramas, %	0,167	0,266	0,401	7,9	Pre-NotRec	Porcentaje de: Nº de muestras no recibidas/Nº total de muestras	0,090	0,190	0,889	Muestras no obtenidas	0,317	6
		(0,155–0,178)	(0,256–0,257)	(0,392–0,410)				(0,060–0,100)	(0,140–0,295)	(0,602–1,470)			
PRE-10	Muestras de sangre total EDTA insuficientes/Hemogramas, %	0,005	0,022	0,062	31,1	Pre-InsV	Porcentaje de: Nº de muestras con volumen insuficiente/Nº total de muestras	0,010	0,030	0,110	Muestra insuficiente	0,165	9
		(0,003–0,007)	(0,018–0,026)	(0,057–0,067)				(0,006–0,011)	(0,022–0,040)	(0,079–0,130)			
PRE-11	Muestras de sangre total EDTA coaguladas/Hemogramas. %	0,087	0,162	0.288	14,1	Pre-Clot	Porcentaje de: Nº de muestras coaguladas/Nº total de muestras en las que se ha comprobado la presencia de coágulos	0,080	0,237	0,402	Muestras coaguladas	0,193	6
		(0,079–0,094)	(0,148–0,175)	(0,272–0,304)				(0,058–0,111)	(0,200–0,270)	(0,350–0,525)			
PRE-12	Rechazos de muestras de coagulación plasma citrato/Pruebas de protrombina, %	0,937	1,887	3,298	10,9		NE				NE		
		(0,842–1,032)	(1,775–1,999)	(2,973–3,623)									
PRE-13	Muestras de de coagulación plasma citrato no recibidas/Pruebas de protrombina, %	0,388	0,743	1,468	15,1	Pre-NotRec	Porcentaje de: Nº de muestras no recibidas/Nº total de muestras	0,090	0,190	0,889	Muestas no obtenidas	0,317	6
		(0,342–0,433)	(0,681–0,805)	(1,348–1,588)				(0,060–0,100)	(0,140–0,295)	(0,602–1,470)			
PRE-14	Muestras de de coagulación plasma citrato con volumen insuficiente/Pruebas de protrombina, %	0,150	0,465	1,230	24,7	Pre-SaAnt	Porcentaje de: Nº de muestras con índice de volumen/anticoagulante inadecuado/Nº total de muestras con anticoagulante	0,070	0,343	0,770	Llenado incorrecto de muestras	0,089	21
		(0,129–0,172)	(0,430–0,500)	(1,108–1,353)				(0,040–0,114)	(0,220–0,420)	(0,690–0,980)			
PRE-15	Muestras de de coagulación plasma citrato coaguladas/Pruebas de protrombina, %	0,042	0,204	0,474	36,8	Pre-Clot	Porcentaje de: Nº de muestras coaguladas/Nº total de muestras con anticoagulante en las que se ha comprobado la presencia de coágulos	0,080	0,237	0,402	Muestras coaguladas	0,193	6
		(0,022–0,062)	(0,175–0,232)	(0,441–0,506)				(0,058–0,111)	(0,200–0,270)	(0,350–0,525)			
PRE-16	Muestras hemolizadas de coagulación plasma citrato/Pruebas de protrombina, %	0,000	0,006	0,095	247,2	Pre-HemR	Porcentaje de: Nº de muestras rechazadas por hemólisis/Nº total de muestras en las que se ha comprobado la presencia de hemólisis	0,049	0,435	0,882	Muestras hemolizadas	0,770	7
		(0,000–0,000)	(0,000–0,011)	(0,069–0,121)				(0,000–0,165)	(0,300–0,500)	(0,587–1,410)			
PRE-17	Muestras de orina no recibidas/Nº total de muestras, %	0,393	0,877	1,370	9,0	Pre-NotRec	Porcentaje de: Nº de muestras no recibidas/Nº total de muestras	0,090	0,190	0,889	Muestras no obtenidas	0,317	6
		(0,337–0,449)	(0,830–0,923)	(1,325–1,415)				(0,060–0,100)	(0,140–0,295)	(0,602–1,470)			
PRE-18	Muestras de suero con índice hemolítico ≥0,5 g/L/Muestras totales de suero con cálculo de índice hemolítico, %	0,758	1,503	2,859	15,3	Pre-HemI	Porcentaje de: Nº de muestras con hemoglobina libre (Hb) > 0,5 g/L (detección automatizada índice de hemólisis)/Nº total de muestras en las que se ha evaluado la hemólisis	0,690	1,810	3,230	NE		
		(0,686–0,831)	(1,408–1,598)	(2,460–3,259)				(0,460–0,970)	(1,490–2,230)	(2,595–3,800)			

IC 95%, intervalo de confianza al 95%; CV, coeficiente de variación; NE, indicador no equivalente. ^a^Coeficiente de variación calculado a partir de los resultados del p50 obtenidos en el periodo 2014–2019.

**Figura 3:**
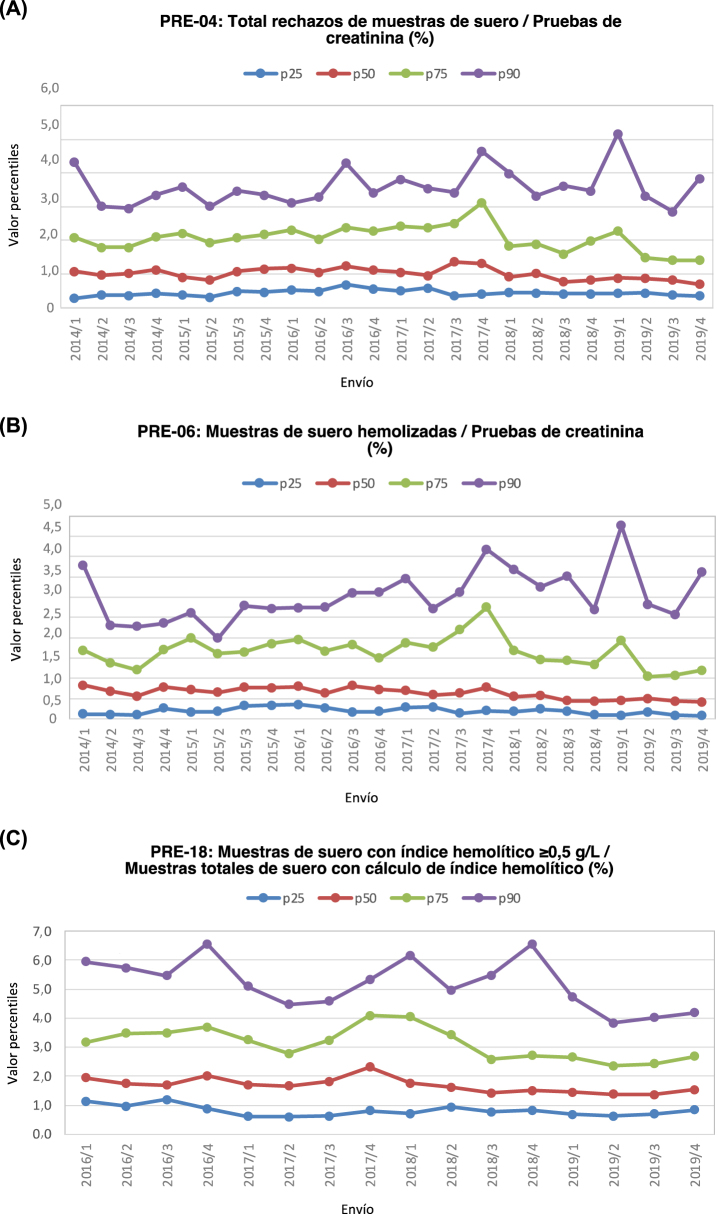
Valores de los percentiles p25, p50, p75 y p90, obtenidos entre el 2014 y 2019 para cada uno de los envíos. (A) PRE-04, (B) PRE-06, (C) PRE-18.

Se observa un descenso significativo entre periodos en lo relativo al valor del p50 para el indicador número total de muestras de suero rechazadas (Figura 3A), asociado a una disminución del p50 del indicador *Rechazos por hemólisis* (Figura 3B y Tabla 2). Las EC se modificaron para ajustarlas al estado del arte de los laboratorios participantes. Este indicador se correspondería con el indicador *Muestras hemolizadas rechazadas* de la IFCC. En 2018, el intervalo de confianza al 95% para la mediana de este indicador fue 0,06–0,87 [13], aunque en este caso, el indicador incluye todos los especímenes y emplea como denominador todas las muestras en las que se ha medido la presencia de hemólisis. En el programa de la SEQC^ML^, las muestras de suero hemolizadas (mediana 0,5%) y de plasma citrato (0,006%) se informaron por separado. Con respecto al programa KIMMS, la media del indicador *Muestra hemolizada* para un periodo de cuatro años (2015–2018) fue de 0,770% con respecto al número total de peticiones [14]. En ambos casos, los resultados son similares a los observados en el programa de la SEQC^ML^ (Tabla 3).

El indicador muestras séricas hemolizadas lo relativo al valor del p50 para el indicadorincluye los rechazos, esto es, aquellas pruebas de las que se ha eliminado al menos un resultado del informe al considerar que se ha producido una interferencia significativa en dicho resultado, con lo que las muestras rechazadas por la presencia de hemólisis (media 0,483%) mostraron una menor prevalencia que las muestras con un índice hemolítico ≥0,5 g/L (1,503%), indicativo del deterioro de la muestra (Figura 3C). El número de muestras séricas con indicador indice hemolítico ≥0,5 g/L superó al de Rechazos por hemólisis*,* ya que la hemólisis de bajo grado puede no causar una interferencia significativa en los resultados del análisis. Tal como ocurre con el indicador *Rechazo por hemólisis,* se observó una tendencia descendente significativa, por lo que se modificaron las EC. Este indicador fue similar al de *Muestras con hemoglobina libre *>0,5 g/L* (detección automática)* de la IFCC*.* En 2018, el intervalo de confianza al 95% para la mediana de este indicador fue de 0,67–2,76 [13], empleando como denominador el número de muestras en las que se determinó la hemólisis (Tabla 3). Con respecto al programa KIMMS, no existe un indicador equivalente*.*

El número de rechazos por hemólisis en el tubo de citrato fue notablemente inferior que en el tubo de suero (0,006% frente al 0,500%), aunque se observa un incremento significativo del indicador entre periodos. Así mismo, se registró un incremento significativo en el número total de rechazos de muestras de sangre total EDTA entre periodos, lo que provocó un cambio en las EC para ambos indicadores. En el caso de las muestras de sangre total EDTA, la principal causa del rechazo preanalítico fue que la muestra estaba coagulada.

El resto de indicadores mostró una evolución considerablemente estable, con tendencia descendente. Destacan los indicadores generales, que permanecieron en niveles muy bajos. El PRE-03 muestras mal identificadas/peticiones totales (%)*,* que podría considerarse un indicador centinela, mostró valores muy bajos (0,009). Este indicador es similar al *p* *orcentaje de:* *n* *úmero de peticiones mal identificadas/número total de peticiones* de la IFCC (0,025%) y al de *d* *iscrepancia de ID del* KIMMS (0,010%). Sin embargo, el número de muestras sin etiquetar (PRE-02 muestras sin etiquetar/peticiones totales (%)) incrementó del 0,009% al 0,014%. Este indicador es similar al *Sin etiquetar* del programa KIMMS, que tiene una EC superior del 0,84%, mientras que no se encuentra un indicador equivalente en el programa de la IFCC.

## Discusión

Existe un interés generalizado en los IC relacionados con las fases extra-analíticas, si bien únicamente un reducido número de laboratorios clínicos recogen con regularidad datos completos al respecto. Se podría considerar que el aumento en el número de participantes en el programa español es un reflejo del creciente interés en estas fases del PAT, aunque el número de participantes sigue estando muy alejado del de los participantes en los programas de calidad de la fase analítica de la SEQC^ML^. Para superar la conocida como “paradoja del indicador de calidad” [25], el WG-LEPS desarrolló un Modelo de Indicadores de Calidad (*MQI,* por sus siglas en inglés) basado en una lista de IC consensuados (disponibles en www.ifcc-mqi.com) [3, 17, 26], y la obtención de los datos informados por los laboratorios clínicos resultó en la definición de EC para la fase preanalítica [25]. En 2017, la SEQC^ML^ calculó las EC con los resultados de los últimos cuatro años de los laboratorios españoles, y que fueron recalculadas con los datos del periodo 2018–2019. El RCPA calculó sus propias EC a partir de los resultados del periodo 2015–2018 aunque, a diferencia de otros programas, los resultados se presentan como medias en lugar de medianas.

Una desventaja importante de los programas de garantía externa de la calidad de la fase preanalítica es la dificultad para comparar resultados, debido a que se aplican fórmulas diferentes para calcular los IC. En este sentido, es necesario lograr la armonización de las mismas. Basándose en los resultados obtenidos en el programa de la SEQC^ML^, los IC calculados a partir de la prueba más frecuente en cada tipo de muestra son los que muestran mejor comparabilidad entre los laboratorios españoles. Además, dado que el número y tipo de errores de las muestras obtenidas en los servicios de urgencias o en las UCI difieren y son más heterogéneos que los observados en las muestras de rutina [27, 28], el programa de la SEQC^ML^ solo incluye datos de las muestras de este tipo, con el fin de evitar este factor de confusión en la interpretación de los resultados. No obstante, algunos de los indicadores son comparables entre programas y se muestran EC en el programa de la SEQC^ML^ muy similares a las de la IFCC, observándose una mayor discrepancia con el programa KIMMS. Los CV también muestran mayor variación, lo que podría deberse a que el periodo de cálculo en el programa es más largo. El programa de la SEQC^ML^ refleja la estabilidad de los indicadores y resulta por tanto útil a la hora de evaluar la evolución de cada laboratorio y comprobar que los datos se están recogiendo correctamente.

El propósito del programa de la SEQC^ML^ es calcular unos indicadores sencillos, que permitan la intercomparación proporcionando una referencia objetiva, a los diferentes laboratorios, estableciendo unas EC basadas en el estado del arte. Esto permite identificar los puntos críticos a lo largo de toda la fase preanalítica, desde la solicitud hasta el manejo y la conservación de la muestra previas al análisis. También permite evaluar las tendencias en el tiempo (positivas o negativas) ya que, aun cuando se cumplen las especificaciones, pueden hallarse evidencias de un empeoramiento. La estabilidad y/o mejora en los indicadores generales (número total de rechazos, muestras no etiquetadas o muestras mal identificadas) podría reflejar la eficacia del programa.

La hemólisis es, con mucho, la principal causa de rechazo de las muestras de suero. Esta puede deberse a errores en la obtención y preparación de la muestra y suele detectarse en la fase analítica. De hecho, en los últimos años se está realizando un gran esfuerzo por mejorar el proceso de extracción de sangre, así como la detección de las muestras hemolizadas, estableciendo medidas que reduzcan su incidencia. El descenso significativo observado en las muestras de suero podría indicar una mejora de las prácticas preanalíticas entre los laboratorios participantes, lo cual es muy positivo. La especificación mínima del programa de la SEQC^ML^ sugiere que los laboratorios deberían mantener el número de rechazos por hemólisis por debajo del 1,4% del número total de muestras de suero. Este indicador es similar al 0,9% establecido en el programa de la IFCC y, en cualquier caso, indica que en multitud de laboratorios se rechazan pruebas en una de cada 100 muestras debido a la presencia de hemólisis. El porcentaje de muestras de suero con un índice hemolítico ≥0,5 g/L se emplea como IC global de la fase preanalítica, ya que indica un deterioro de la muestra durante alguno de los procesos preanalíticos (obtención, transporte o preparación). La mejora en dicho IC también podría indicar una mayor concienciación sobre la importancia de la fase preanalítica en el laboratorio. En este caso, los resultados del programa de la SEQC^ML^ y de la IFCC sugieren que los laboratorios deberían mantener un porcentaje de muestras hemolizadas (índice hemolítico ≥0,5 g/L) inferior al 3%.

Considerando el p75 (especificaciones mínimas) y el tipo de espécimen, el IC de las muestras de plasma citrato para coagulación son las que arrojan peores resultados, por lo que los laboratorios deberían adoptar medidas para reducir el número de muestras insuficientes, coaguladas y hemolizadas. Por otro lado, el empeoramiento de los resultados podría reflejar una mayor prestación de los nuevos analizadores de hemostasia, que son capaces de detectar un mayor número de parámetros preanalíticos, tal como ocurrió hace algunos años con las muestras de suero y los analizadores de bioquímica, capaces de medirlos índices hemolíticos, de ictericia y turbidez. Cualesquiera sean las razones, es responsabilidad de los laboratorios mejorar este IC. De acuerdo con los resultados obtenidos, también se debería reducir el número de muestras coaguladas de sangre total EDTA.

En los programas se obtuvieron resultados muy similares en cuanto a las especificaciones. En cuanto a los CV, son superiores a los del programa KIMMS, aunque esto podría deberse al método de cálculo, ya que en el caso del programa de la SEQC^ML^, no se han excluido posibles valores atípicos (*outliers*).

En este estudio, para calcular las EC se emplearon los resultados de los IC obtenidos entre 2014 y 2017, y se compararon posteriormente con las obtenidas en el periodo 2018–2019, con el fin de identificar los procesos que se deben mejorar de manera prioritaria. En el año próximo, se recalcularán dichas EC, en caso de que se obtengan mejores resultados para el periodo 2020–2021, se considerará la posibilidad de cambiar las especificaciones. No obstante, la mejora general de los resultados obtenidos en el periodo 2018–2019 refleja la conciencia que han adquirido los laboratorios clínicos sobre la importancia de la fase preanalítica y los esfuerzos realizados para mejorarla. En el futuro, debido a los rápidos avances en las pruebas diagnósticas y en los tipos de muestras empleados en los laboratorios (por ejemplo, muestras para las pruebas realizadas en ADN circulante libre), puede que sea necesario revisar la validez de los IC actuales e introducir nuevos. En este sentido, la Comisión de Calidad Extra-analítica de la SEQC^ML^ ha incluido un nuevo IC en el programa de 2021: número de rechazos por muestras de suero contaminadas por fluidos de infusión/Número de pruebas de creatinina (%). Además, la mejora en las herramientas de los SIL permitirá simplificar la obtención y gestión de datos y junto con el incremento de participantes en el programa, también podrían permitir incluir los resultados obtenidos en los servicios de urgencias.

Una de las principales limitaciones del presente estudio es el reducido número de resultados, debido al número limitado de laboratorios participantes (72 para el periodo 2018–2019), que no es comparable con el enorme volumen de datos empleado en los programas de calidad de la fase analítica (unos 600 laboratorios en 2019 en el programa EQA de SUERO de la SEQC^ML^, programa con mayor número de participantes), así como la no exclusión de *outliers*. Estas limitaciones podrían comprometer la robustez de las especificaciones calculadas.

Otra desventaja de este tipo de programa, diferente de los programas para las fases analíticas, es que, en caso de no cumplirse las especificaciones, las medidas que se deben adoptar para resolver el problema y mejorar los resultados de los IC podrían no estar claras. Se debería realizar un análisis de los riesgos asociados a cada situación, lo que resultaría útil a la hora de priorizar los puntos a intervenir. Una vez aplicadas las medidas oportunas, la comparación de los nuevos IC obtenidos con las especificaciones propuestas permitiría verificar la eficacia de las medidas implementadas.

En conclusión, los IC y las especificaciones preanalíticas ayudan a identificar la existencia de errores y evaluar la eficacia de las acciones correctivas adoptadas, aunque por sí mismas, no controlan procesos o identifican las causas de los errores, es responsabilidad de todas las sociedades científicas, así como de los laboratorios, realizar un seguimiento e implementar las medidas necesarias. El programa de garantía externa de calidad de la fase preanalítica de la SEQC^ML^ ofrece una fórmula fiable para calcular los IC, y las especificaciones obtenidas son una herramienta útil a la hora de realizar estudios comparativos, así como para identificar procesos cuya mejora es prioritaria en los laboratorios clínicos.

## Supplementary Material

Supplementary Material

## Material Suplementario

La versión en línea de este artículo ofrece material suplementario (https://doi.org/10.1515/almed-2022-0036).
