# Supplementary material for: Evaluación de 18 indicadores de calidad del Programa de Garantía Externa de la Calidad de Preanalítica de la Sociedad Española de Medicina de Laboratorio (SEQCML)
Source: Adv Lab Med. 2022 Jun 8;3(2):188–200. [Article in Spanish] doi: 10.1515/almed-2022-0036 (PMC10197359; doi:10.1515/almed-2022-0036)
Supplement: Supplementary file 1 — Supplementary Material [file j_almed-2022-0036_suppl.docx]

**Tabla S1.** Media, desviación estándar (DE), coeficiente de variación (CV) y número de envíos (n) para cada percentil (p25, p50, p75 y p90) agrupados por periodos: 2019-2014, 2014-2017 y 2018-2019, para cada indicador de calidad (IC). NC: no calculado.

| **p25** | **2014-2019** | | | | **2014-2017** | | | | **2018-2019** | | | |
| --- | --- | --- | --- | --- | --- | --- | --- | --- | --- | --- | --- | --- |
| **IC** | **media** | **DE** | **CV%** | **n** | **media** | **DE** | **CV%** | **n** | **media** | **DE** | **CV%** | **n** |
| PRE-01 | 1,403 | 0,177 | 12,64 | 24 | 1,409 | 0,189 | 13,40 | 16 | 1,393 | 0,164 | 11,77 | 8 |
| PRE-02 | 0,000 | 0,000 | NC | 24 | 0,000 | 0,000 | NC | 16 | 0,000 | 0,000 | NC | 8 |
| PRE-03 | 0,001 | 0,001 | 139,91 | 24 | 0,001 | 0,001 | 119,96 | 16 | 0,000 | 0,001 | 198,41 | 8 |
| PRE-04 | 0,441 | 0,092 | 20,93 | 24 | 0,452 | 0,110 | 24,36 | 16 | 0,418 | 0,033 | 7,98 | 8 |
| PRE-05 | 0,103 | 0,023 | 22,81 | 24 | 0,102 | 0,027 | 26,77 | 16 | 0,104 | 0,014 | 13,69 | 8 |
| PRE-06 | 0,197 | 0,084 | 42,97 | 24 | 0,222 | 0,084 | 37,98 | 16 | 0,146 | 0,062 | 42,30 | 8 |
| PRE-07 | 0,004 | 0,004 | 94,84 | 24 | 0,004 | 0,004 | 99,71 | 16 | 0,003 | 0,003 | 82,20 | 8 |
| PRE-08 | 0,297 | 0,035 | 11,72 | 24 | 0,288 | 0,035 | 12,05 | 16 | 0,317 | 0,028 | 8,69 | 8 |
| PRE-09 | 0,145 | 0,029 | 19,87 | 24 | 0,134 | 0,027 | 20,49 | 16 | 0,167 | 0,017 | 9,98 | 8 |
| PRE-10 | 0,005 | 0,004 | 74,55 | 24 | 0,005 | 0,004 | 81,75 | 16 | 0,005 | 0,003 | 62,34 | 8 |
| PRE-11 | 0,077 | 0,020 | 25,73 | 24 | 0,072 | 0,022 | 29,93 | 16 | 0,087 | 0,011 | 13,12 | 8 |
| PRE-12 | 0,853 | 0,175 | 20,56 | 24 | 0,811 | 0,181 | 22,30 | 16 | 0,937 | 0,137 | 14,65 | 8 |
| PRE-13 | 0,376 | 0,073 | 19,36 | 24 | 0,371 | 0,078 | 20,96 | 16 | 0,388 | 0,065 | 16,89 | 8 |
| PRE-14 | 0,124 | 0,065 | 51,86 | 24 | 0,112 | 0,074 | 65,95 | 16 | 0,150 | 0,031 | 20,47 | 8 |
| PRE-15 | 0,024 | 0,028 | 116,03 | 24 | 0,015 | 0,024 | 155,00 | 16 | 0,042 | 0,029 | 69,29 | 8 |
| PRE-16 | 0,000 | 0,000 | NC | 24 | 0,000 | 0,000 | NC | 16 | 0,000 | 0,000 | NC | 8 |
| PRE-17 | 0,365 | 0,077 | 21,16 | 24 | 0,351 | 0,074 | 21,11 | 16 | 0,393 | 0,081 | 20,50 | 8 |
| PRE-18 | 0,805 | 0,183 | 22,73 | 16 | 0,852 | 0,236 | 27,72 | 8 | 0,758 | 0,105 | 13,79 | 8 |

| **p50** | **2014-2019** | | | | **2014-2017** | | | | **2018-2019** | | | |
| --- | --- | --- | --- | --- | --- | --- | --- | --- | --- | --- | --- | --- |
| **IC** | **media** | **DE** | **CV%** | **n** | **media** | **DE** | **CV%** | **n** | **media** | **DE** | **CV%** | **n** |
| PRE-01 | 2,238 | 0,192 | 8,58 | 24 | 2,266 | 0,219 | 9,66 | 16 | 2,182 | 0,115 | 5,26 | 8 |
| PRE-02 | 0,010 | 0,006 | 59,22 | 24 | 0,009 | 0,006 | 68,46 | 16 | 0,014 | 0,005 | 37,41 | 8 |
| PRE-03 | 0,010 | 0,003 | 33,81 | 24 | 0,010 | 0,003 | 31,50 | 16 | 0,009 | 0,003 | 38,25 | 8 |
| PRE-04 | 1,011 | 0,171 | 16,92 | 24 | 1,091 | 0,143 | 13,07 | 16 | 0,853 | 0,097 | 11,35 | 8 |
| PRE-05 | 0,195 | 0,025 | 12,87 | 24 | 0,188 | 0,026 | 13,81 | 16 | 0,210 | 0,016 | 7,76 | 8 |
| PRE-06 | 0,640 | 0,135 | 21,16 | 24 | 0,718 | 0,083 | 11,60 | 16 | 0,483 | 0,057 | 11,86 | 8 |
| PRE-07 | 0,036 | 0,009 | 25,97 | 24 | 0,037 | 0,010 | 27,27 | 16 | 0,035 | 0,009 | 24,51 | 8 |
| PRE-08 | 0,493 | 0,060 | 12,15 | 24 | 0,481 | 0,064 | 13,27 | 16 | 0,519 | 0,044 | 8,58 | 8 |
| PRE-09 | 0,254 | 0,020 | 7,88 | 24 | 0,248 | 0,020 | 8,24 | 16 | 0,266 | 0,013 | 5,06 | 8 |
| PRE-10 | 0,025 | 0,008 | 31,07 | 24 | 0,026 | 0,008 | 32,39 | 16 | 0,022 | 0,005 | 24,69 | 8 |
| PRE-11 | 0,154 | 0,022 | 14,15 | 24 | 0,150 | 0,022 | 14,93 | 16 | 0,162 | 0,019 | 11,96 | 8 |
| PRE-12 | 1,795 | 0,195 | 10,88 | 24 | 1,748 | 0,198 | 11,35 | 16 | 1,887 | 0,162 | 8,58 | 8 |
| PRE-13 | 0,803 | 0,121 | 15,09 | 24 | 0,833 | 0,126 | 15,14 | 16 | 0,743 | 0,089 | 12,00 | 8 |
| PRE-14 | 0,441 | 0,109 | 24,72 | 24 | 0,430 | 0,129 | 30,01 | 16 | 0,465 | 0,050 | 10,85 | 8 |
| PRE-15 | 0,165 | 0,061 | 36,82 | 24 | 0,146 | 0,061 | 41,71 | 16 | 0,204 | 0,041 | 20,12 | 8 |
| PRE-16 | 0,002 | 0,005 | 247,16 | 24 | 0,000 | 0,001 | 400,00 | 16 | 0,006 | 0,008 | 140,28 | 8 |
| PRE-17 | 0,828 | 0,075 | 9,03 | 24 | 0,804 | 0,068 | 8,48 | 16 | 0,877 | 0,067 | 7,62 | 8 |
| PRE-18 | 1,682 | 0,257 | 15,26 | 16 | 1,861 | 0,222 | 11,94 | 8 | 1,503 | 0,137 | 9,09 | 8 |

| **p75** | **2014-2019** | | | | **2014-2017** | | | | **2018-2019** | | | |
| --- | --- | --- | --- | --- | --- | --- | --- | --- | --- | --- | --- | --- |
| **IC** | **media** | **DE** | **CV%** | **n** | **media** | **DE** | **CV%** | **n** | **media** | **DE** | **CV%** | **n** |
| PRE-01 | 3,253 | 0,325 | 9,98 | 24 | 3,272 | 0,343 | 10,47 | 16 | 3,215 | 0,303 | 9,44 | 8 |
| PRE-02 | 3,253 | 0,325 | 9,98 | 24 | 3,272 | 0,343 | 10,47 | 16 | 3,215 | 0,303 | 9,44 | 8 |
| PRE-03 | 0,044 | 0,011 | 25,55 | 24 | 0,041 | 0,012 | 28,15 | 16 | 0,049 | 0,009 | 18,07 | 8 |
| PRE-04 | 0,027 | 0,011 | 39,76 | 24 | 0,030 | 0,012 | 38,43 | 16 | 0,020 | 0,003 | 16,85 | 8 |
| PRE-05 | 2,062 | 0,389 | 18,85 | 24 | 2,224 | 0,320 | 14,41 | 16 | 1,737 | 0,309 | 17,76 | 8 |
| PRE-06 | 0,335 | 0,040 | 12,01 | 24 | 0,328 | 0,041 | 12,62 | 16 | 0,350 | 0,036 | 10,22 | 8 |
| PRE-07 | 1,659 | 0,379 | 22,83 | 24 | 1,791 | 0,349 | 19,49 | 16 | 1,397 | 0,304 | 21,75 | 8 |
| PRE-08 | 0,108 | 0,020 | 18,64 | 24 | 0,107 | 0,022 | 20,61 | 16 | 0,111 | 0,017 | 15,19 | 8 |
| PRE-09 | 0,767 | 0,086 | 11,24 | 24 | 0,766 | 0,103 | 13,46 | 16 | 0,769 | 0,041 | 5,29 | 8 |
| PRE-10 | 0,410 | 0,040 | 9,88 | 24 | 0,414 | 0,049 | 11,79 | 16 | 0,401 | 0,012 | 3,11 | 8 |
| PRE-11 | 0,066 | 0,010 | 15,85 | 24 | 0,068 | 0,011 | 16,61 | 16 | 0,062 | 0,008 | 12,47 | 8 |
| PRE-12 | 0,264 | 0,038 | 14,26 | 24 | 0,252 | 0,038 | 15,11 | 16 | 0,288 | 0,023 | 8,10 | 8 |
| PRE-13 | 3,008 | 0,415 | 13,81 | 24 | 2,863 | 0,307 | 10,74 | 16 | 3,298 | 0,469 | 14,22 | 8 |
| PRE-14 | 1,533 | 0,135 | 8,80 | 24 | 1,565 | 0,103 | 6,58 | 16 | 1,468 | 0,173 | 11,78 | 8 |
| PRE-15 | 1,096 | 0,209 | 19,07 | 24 | 1,028 | 0,194 | 18,89 | 16 | 1,230 | 0,177 | 14,40 | 8 |
| PRE-16 | 0,412 | 0,089 | 21,67 | 24 | 0,381 | 0,090 | 23,68 | 16 | 0,474 | 0,047 | 9,97 | 8 |
| PRE-17 | 0,083 | 0,042 | 50,17 | 24 | 0,077 | 0,044 | 56,47 | 16 | 0,095 | 0,037 | 39,36 | 8 |
| PRE-18 | 1,363 | 0,070 | 5,13 | 24 | 1,359 | 0,074 | 5,45 | 16 | 1,370 | 0,065 | 4,74 | 8 |

| **p90** | **2014-2019** | | | | **2014-2017** | | | | **2018-2019** | | | |
| --- | --- | --- | --- | --- | --- | --- | --- | --- | --- | --- | --- | --- |
| **IC** | **media** | **DE** | **CV%** | **n** | **media** | **DE** | **CV%** | **n** | **media** | **DE** | **CV%** | **n** |
| PRE-01 | 4,666 | 0,445 | 9,53 | 24 | 4,695 | 0,465 | 9,90 | 16 | 4,608 | 0,426 | 9,25 | 8 |
| PRE-02 | 0,091 | 0,021 | 23,29 | 24 | 0,086 | 0,021 | 24,56 | 16 | 0,102 | 0,018 | 17,66 | 8 |
| PRE-03 | 0,060 | 0,027 | 44,12 | 24 | 0,068 | 0,029 | 43,42 | 16 | 0,045 | 0,007 | 16,44 | 8 |
| PRE-04 | 3,588 | 0,560 | 15,60 | 24 | 3,534 | 0,500 | 14,15 | 16 | 3,696 | 0,688 | 18,61 | 8 |
| PRE-05 | 0,657 | 0,116 | 17,69 | 24 | 0,678 | 0,117 | 17,19 | 16 | 0,616 | 0,111 | 18,04 | 8 |
| PRE-06 | 3,039 | 0,650 | 21,38 | 24 | 2,876 | 0,573 | 19,92 | 16 | 3,365 | 0,708 | 21,05 | 8 |
| PRE-07 | 0,239 | 0,067 | 27,89 | 24 | 0,232 | 0,070 | 30,13 | 16 | 0,252 | 0,061 | 24,43 | 8 |
| PRE-08 | 1,117 | 0,111 | 9,94 | 24 | 1,143 | 0,126 | 11,02 | 16 | 1,066 | 0,045 | 4,25 | 8 |
| PRE-09 | 0,709 | 0,133 | 18,73 | 24 | 0,751 | 0,142 | 18,86 | 16 | 0,624 | 0,049 | 7,90 | 8 |
| PRE-10 | 0,135 | 0,035 | 26,17 | 24 | 0,146 | 0,037 | 25,25 | 16 | 0,113 | 0,018 | 16,36 | 8 |
| PRE-11 | 0,447 | 0,085 | 18,98 | 24 | 0,443 | 0,099 | 22,29 | 16 | 0,456 | 0,052 | 11,31 | 8 |
| PRE-12 | 4,979 | 1,015 | 20,39 | 24 | 4,734 | 0,961 | 20,29 | 16 | 5,471 | 0,998 | 18,24 | 8 |
| PRE-13 | 2,329 | 0,455 | 19,53 | 24 | 2,273 | 0,497 | 21,86 | 16 | 2,441 | 0,359 | 14,70 | 8 |
| PRE-14 | 2,228 | 0,676 | 30,35 | 24 | 2,028 | 0,580 | 28,60 | 16 | 2,629 | 0,712 | 27,06 | 8 |
| PRE-15 | 0,855 | 0,160 | 18,72 | 24 | 0,822 | 0,152 | 18,52 | 16 | 0,920 | 0,165 | 17,94 | 8 |
| PRE-16 | 0,442 | 0,221 | 50,01 | 24 | 0,384 | 0,208 | 54,30 | 16 | 0,557 | 0,210 | 37,74 | 8 |
| PRE-17 | 1,968 | 0,205 | 10,41 | 24 | 2,017 | 0,214 | 10,59 | 16 | 1,869 | 0,152 | 8,15 | 8 |
| PRE-18 | 5,194 | 0,859 | 16,54 | 16 | 5,400 | 0,693 | 12,83 | 8 | 4,988 | 1,003 | 20,10 | 8 |
